# An integrated analysis of human myeloid cells identifies gaps in *in vitro* models of *in vivo* biology

**DOI:** 10.1016/j.stemcr.2021.04.010

**Published:** 2021-05-13

**Authors:** Nadia Rajab, Paul W. Angel, Yidi Deng, Jennifer Gu, Vanta Jameson, Mariola Kurowska-Stolarska, Simon Milling, Chris M. Pacheco, Matt Rutar, Andrew L. Laslett, Kim-Anh Lê Cao, Jarny Choi, Christine A. Wells

**Affiliations:** 1 The Centre for Stem Cell Systems, Faculty of Medicine, Dentistry and Health Sciences, The University of Melbourne, 30 Royal Parade, Parkville, VIC 3010, Australia; 2 CSIRO Synthetic Biology Future Science Platform, Australia; 3 Melbourne Integrative Genomics, School of Mathematics and Statistics, Faculty of Science, The University of Melbourne, 30 Royal Parade, Parkville, VIC 3010, Australia; 4 Melbourne Cytometry Platform (MBC Node), Faculty of Medicine, Dentistry and Health Sciences, University of Melbourne, Melbourne, Australia; 5 The Institute of Infection, Immunity and Inflammation, Research into Inflammatory Arthritis Centre *‘Versus Arthritis’* (RACE), University of Glasgow, Glasgow, UK; 6 CSIRO Manufacturing, Clayton, VIC 3168, Australia; 7 Australian Regenerative Medicine Institute, Monash University, Melbourne, VIC 3800, Australia

**Keywords:** monocyte, macrophage, dendritic cell, microglia, Kupffer cell, tissue-resident macrophage, hematopoietic progenitor, monocyte-derived macrophage, pluripotent stem cell–derived macrophage, transcriptome

## Abstract

The Stemformatics myeloid atlas is an integrated transcriptome atlas of human macrophages and dendritic cells that systematically compares freshly isolated tissue-resident, cultured, and pluripotent stem cell–derived myeloid cells. Three classes of tissue-resident macrophage were identified: Kupffer cells and microglia; monocyte-associated; and tumor-associated macrophages. Culture had a major impact on all primary cell phenotypes. Pluripotent stem cell–derived macrophages were characterized by atypical expression of collagen and a highly efferocytotic phenotype. Myeloid subsets, and phenotypes associated with derivation, were reproducible across experimental series including data projected from single-cell studies, demonstrating that the atlas provides a robust reference for myeloid phenotypes. Implementation in Stemformatics.org allows users to visualize patterns of sample grouping or gene expression for user-selected conditions and supports temporary upload of your own microarray or RNA sequencing samples, including single-cell data, to benchmark against the atlas.

## Highlights

• A reference transcriptome atlas for human macrophage biology
• Culture alters primary myeloid phenotypes
• Pluripotent stem cell–derived macrophages retain a common stromal signature
• FLT3L-derived cord blood DCs lack expression of key pattern recognition receptors

## Graphical abstract

**Figure:**
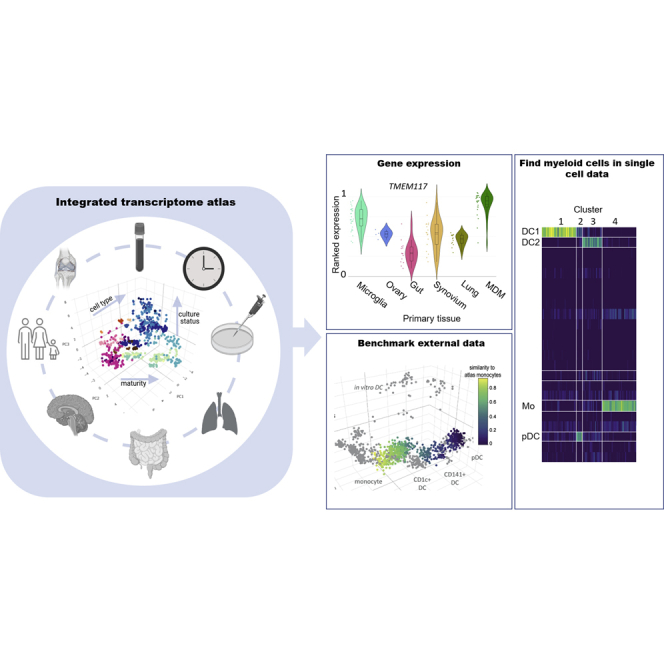

## Teaser

In this article, Wells and colleagues describe an integrated myeloid transcriptome atlas. Analysis resulted in tissue-resident populations being categorized into three broad classes, with culture also found to impact primary cell phenotypes. Pluripotent stem cell–derived cells were compared with their *in vivo* counterparts, differing in maturation, efferocytosis capacity, and ectopic expression of extracellular matrix genes.

## Introduction

Macrophages are innate immune cells that are resident in every tissue, have homeostatic roles, and respond to infection or injury. The distinct functional roles of macrophages are reflected in their transcriptional phenotypes: tissue atlases of mouse macrophages, for example, have given great insight into their complexity and heterogeneity (Gautier et al., 2012; Gosselin et al., 2017). Individual transcriptome studies have revealed the importance of the environment on human macrophage phenotype and function (reviewed by Huang and Wells, 2014), but these lack coherence with respect to the distinctiveness or similarities of human myeloid subsets.

Much of our understanding of macrophage biology, including many of the molecular mechanisms of innate immune signaling, have arisen from mouse gene knockout studies. However, cross-species comparisons of immune cells highlight differences between mouse and human. These include the glycolytic switch associated with metabolic reprogramming in activated mouse macrophages (Vijayan et al., 2019), divergent patterns of pathogen receptor expression (Vijayan et al., 2012), and transcriptional responses to innate immune stimuli (Schroder et al., 2012). Cross-species comparisons are further hampered by the absence of population-level immune-activation maps, with most mouse studies in macrophage biology conducted on a limited number of inbred lines. Although databases such as BloodSpot (Bagger et al., 2016) and Haematlas (Watkins et al., 2009) provide a useful snapshot of gene expression of human blood types, these lack depth with regard to tissue representation, or activating stimuli.

The need for improved molecular models of primary human cells is evident from the rising popularity of single-cell transcriptomic atlases, exemplified by the human cell atlas consortium (Hay et al., 2018; Regev et al., 2017). However, unbiased profiling of cells also requires computational predictions of cell identity, raising further questions about how best to accurately identify immune cell populations resident in tissues, and discriminate these from circulating or infiltrating peripheral blood cells. The isolation, and identification of tissue-resident myeloid cells can be particularly fraught if populations are rare or hard to isolate using enzymatic or other dissociation methods. These procedures can alter myeloid transcriptomes (Gosselin et al., 2017), resulting in underrepresentation or phenotypic ambiguity of resident macrophages in single-cell maps of a tissue. It might be argued that human macrophages suffer from an identity crisis, relying on equivalency to laboratory models of human macrophage biology such as *ex vivo* culture of monocyte-derived macrophages, which may not be appropriate as a benchmark for specialized tissue functions.

Pluripotent stem cells provide new opportunities to model tissue residency, disease phenotypes and activation status of human macrophages (reviewed by Lee et al., 2018; Rajab et al., 2018). However, the anatomic context or ontogeny of PSC-derived cells is still not well understood, nor their capacity to model specialized myeloid behaviors within a tissue niche. Consequently, comparisons of new models of human macrophage biology rely on *ad hoc* comparisons that do not adequately represent the diversity of possible macrophage phenotypes. Here, we describe an integrated myeloid transcriptome atlas to identify, benchmark, and analyze human myeloid subpopulations from *ex vivo*, *in vivo,* and *in vitro* sources. The interactive atlas is available at https://www.stemformatics.org/atlas/myeloid.

## Results

### A reference atlas for human myeloid biology

**Figure 1:**
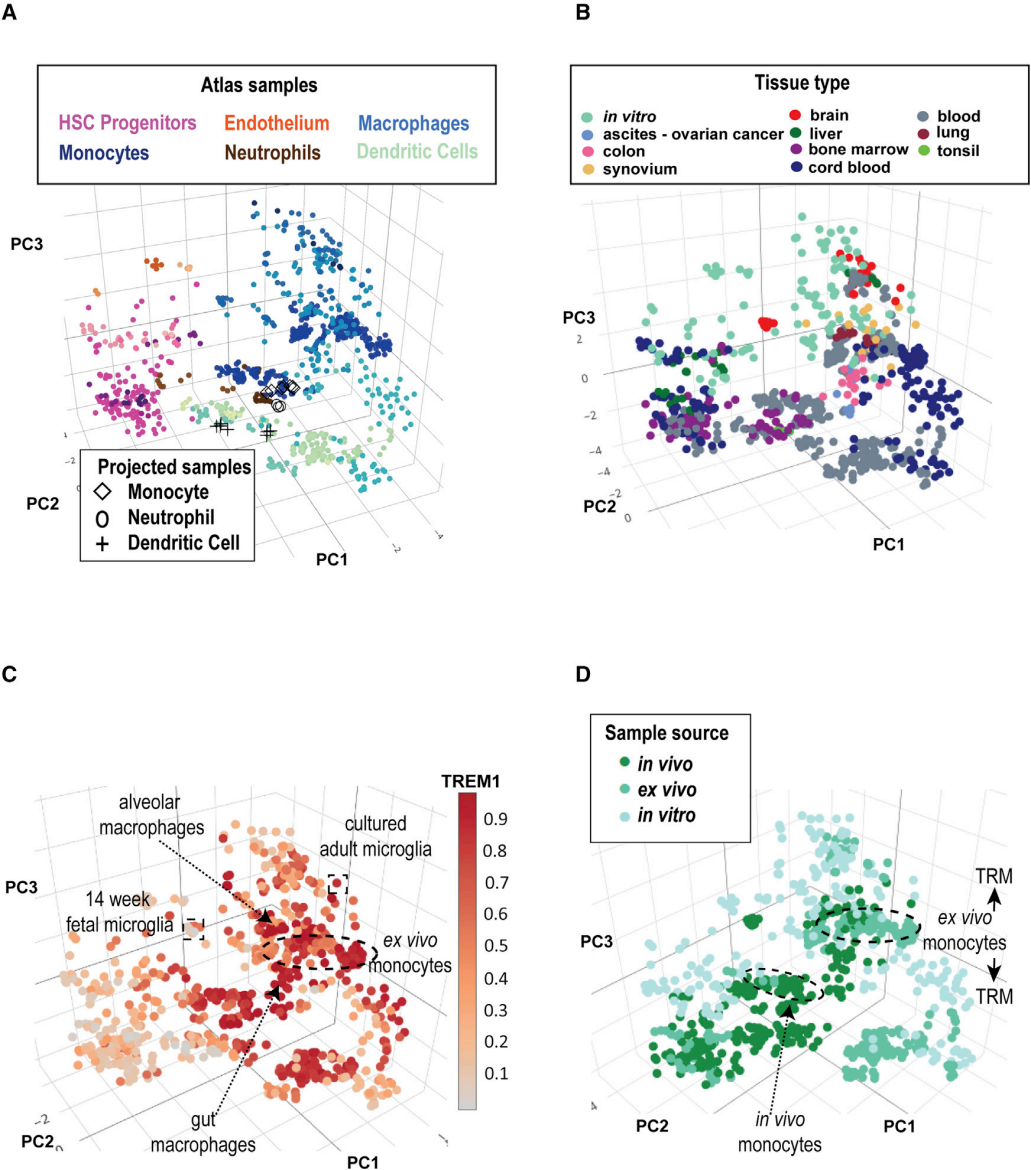
A reference atlas for human myeloid biology (A) Stemformatics myeloid atlas with samples colored by cell type. Navy blue - monocytes, blue - macrophages, aqua - dendritic cells, dark green - CD141 + DC, light green - CD1c + DC, yellow - pDC, brown - granulocytes, pink stem and progenitor cells, hemogenic endothelium. Validation with Haemopedia RNA-seq myeloid samples: diamond shape - monocytes, circle - granulocytes, cross - DC. (B) Stemformatics myeloid atlas with samples colored by tissue type. (C) Stemformatics myeloid Atlas colored by ranked expression of *TREM1*. (Scale bar: high ranked expression [dark red] to low ranked expression [gray]). (D) Stemformatics myeloid atlas colored by culture status (sample source). Positioning of *ex vivo* and *in vivo* monocytes highlighted alongside location of tissue-resident macrophages (TRM) above and below *ex vivo* monocyte positioning. See also Table 1, Figure S1, Tables S1 and S2.

We first compiled a reference transcriptional atlas (Figure 1A and Table S1) from 44 studies and ~900 samples representing monocytes, tissue-resident, *ex vivo* and *in vitro*–derived macrophages, and dendritic cells (DCs). Samples were curated with respect to phenotype, source, and culture method (Choi et al., 2019a). The resulting atlas led to reproducible clustering of distinct myeloid subsets (Figures 1 and S1A). The reproducibility of these clusters was validated by alignment of an independent RNA sequencing (RNA-seq) dataset of well-annotated blood cell types from Haemopedia (Choi et al., 2019b). Variables such as progenitor source (Figure 1B), tissue of origin, disease status, culture status, and activating stimuli can be viewed side-by-side with patterns of gene expression (Figures 1C and S1B). Gene expression is viewed as a color scale across the atlas, or as a summary graph of conditions chosen by the user. This allows users to assess markers of myeloid subsets, such as the expression of *TREM1* on monocytes, monocyte-derived macrophages (MDMs), alveolar macrophages and CD1c + DCs (DC2) (Figure 1B and Table 1), understand the impact of culture or disease status on marker expression, and discover combinations of variables that influence myeloid phenotypes. External data can be projected onto the atlas by users, including single-cell datasets such as PSC-microglia samples from (Mancuso et al., 2019) (Figure S1C).

**Table 1:** Atlas inclusion of tissue-resident monocytes and macrophages

Tissue	Tier 1 (sample no.)	Tier 2	Cell type
Blood	*in vivo* (64) *ex vivo* (162)	myeloid	monocyte
Cord blood	*ex vivo* (51)	myeloid	monocyte
Gut	*in vivo (15)*	myeloid	macrophage
Synovium	*in vivo (18)*	myeloid	macrophage
Brain	*in vivo* (10) *ex vivo* (21)	myeloid	microglia
Lung	*in vivo* (14)	myeloid	macrophage
Liver	*ex vivo* (5)	myeloid	Kupffer cell
Ovarian tumor	*in vivo* (4)	myeloid	macrophage

### Three broad classes of tissue-resident macrophage

Tissue-resident macrophages form three broad clusters on the atlas, with Kupffer cells and microglia aligning to PSC-derived cells (Figure 1). Alveolar macrophages and synovial macrophages are most closely associated with cultured monocytes, and tumor-associated macrophages (TAM) isolated from resected colon or breast ascites occupy a broad niche on the atlas between DCs and cultured monocytes (Figures 1A and 1B). The top genes associated with the TAM group included *CD1d, CD1e*, and *CD207* (Langerin), which may reflect the interactions in the tissues surveyed between macrophages and iNKT (Cortesi et al., 2018). Microglia and Kupffer cells shared high expression of *TMEM117, SEZ6L2*, and *DCDC1*; and lung and synovial macrophages shared expression of enzymes *AKR1C3, PCOLCE2*, and chemokine *CXCL3* (Table S2).

The difficulty of isolating tissue-resident macrophages from healthy human tissue is evident from the spread of tissue-resident macrophages in comparison to tissue-resident DCs (Figures 1A and 1B), noting that several of the macrophage datasets were obtained through surgical biopsies from patients with inflammatory disease. This spread could not be attributed to the method of cell isolation, as it was seen even within the same dataset. We were not able to extract sufficient experimental detail from contributing studies to determine whether specific dissociation parameters were contributors to this expression variation, although others have shown that isolation of primary tissue-resident macrophages can result in alterations in phenotype (Gosselin et al., 2017).

### DC differentiated from cord blood lack an *in vivo* equivalent

**Figure 2:**
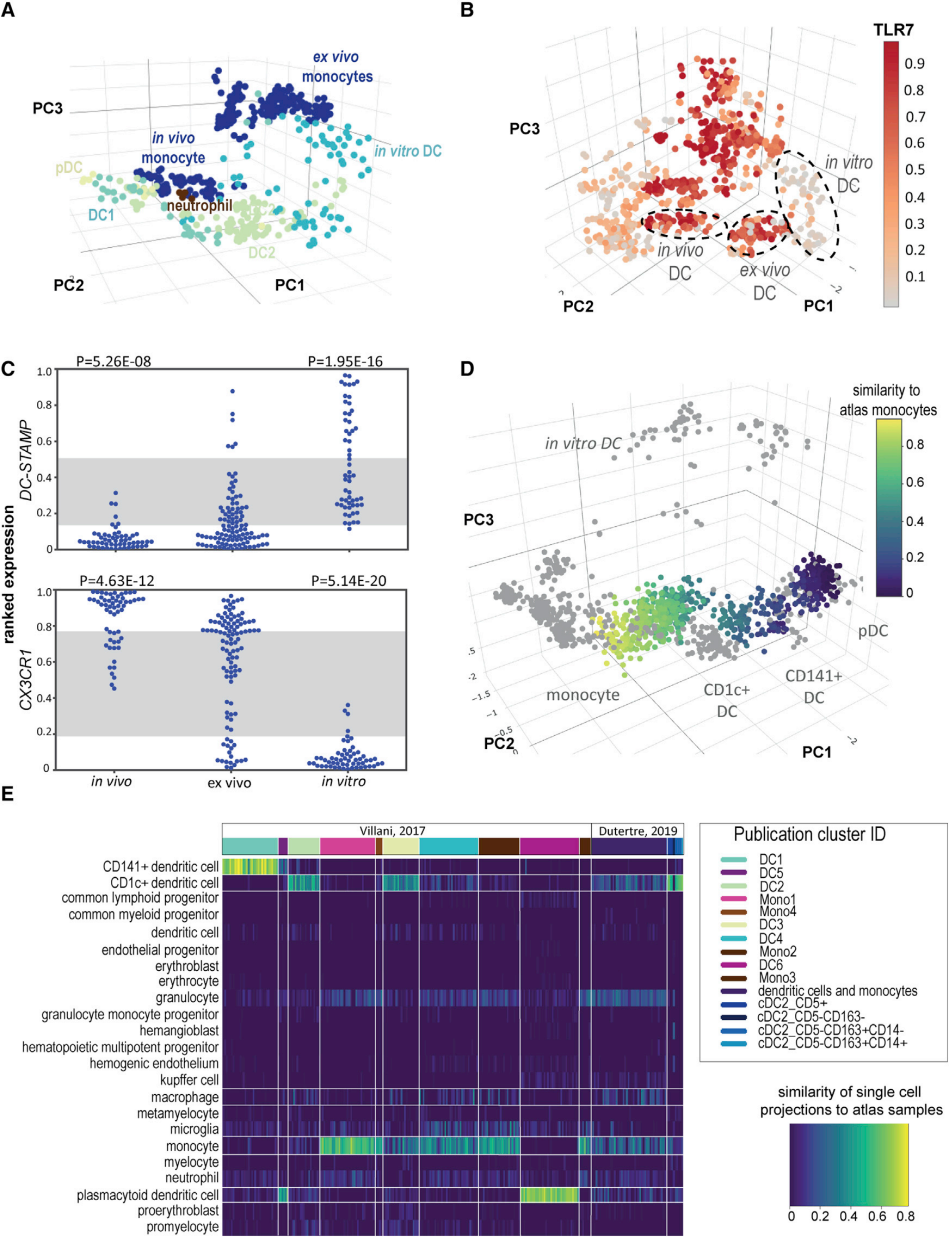
Cultured and *in vitro*–derived DCs do not capture aspects *in vivo* myeloid biology (A) Dendritic cell (DC) subsets displayed in the atlas – aqua *in vitro* (cord blood derived) DC, dark green DC1 (CD141+), light green DC2 (CD1c+), yellow - pDC, brown neutrophils. (B) Atlas colored by ranked expression of *TLR7* (Scale bar: high ranked expression [dark red] to low ranked expression [gray]). Location of *in vivo*, *ex vivo,* and *in vitro* DCs highlighted. (C) Ranked expression (y axis) of receptor *CX3CR1* and cell-fusion protein *DC-STAMP in vivo* DCs (n = 57), *ex vivo* DCs (n = 105), and *in vitro*–derived DCs (n = 57). Gray stripe indicates variance attributable to platform. p value: Mann-Whitney-Wilcoxon rank-sum test. (D) Single-cell projections of Villani et al. (2017) and Dutertre et al. (2019) samples onto atlas. Similarity score high (yellow) to low (blue). (F) Heatmap derived from Capybara analysis of Villani et al. (2017) and Dutertre et al. (2019) samples compared with myeloid cell types. Color gradients reflect similarity of single-cell clusters to atlas cell types (dark least similar, to light most similar). See also Figure S2 and Table S3.

Circulating and tissue-resident DCs occupy a distinct transcriptional niche from monocytes or macrophages. *In vitro* differentiated DCs, expanded from cord blood progenitors with FLT3L, do not resemble *in vivo* conventional DCs (DC1 or DC2; Figure 2A). The hallmark of *in vitro* DCs is low expression of receptors such as *CX3CR1*, *IL18R1,* and *TLR7* (Figures 2B and 2C and Table S3). Other molecules, such as the cell-fusion protein *DC-STAMP* are gained in culture (Figure 2C and Table S3). These cells are also closely associated with MDMs, which may contribute to some of the confusion in the literature about their ontogeny. It is unlikely that this is due to the cord blood origins of the majority of these datasets, as CD45+ cells isolated from cord blood engrafted into humanized mice are also included in the atlas (Minoda et al., 2017) and these recapitulate *in vivo* DC1 and DC2 phenotypes.

We did observe distinct grouping of DC1, DC2, and plasmacytoid DCs (pDC) subsets. To further evaluate these, we projected two single-cell RNA-seq datasets describing blood monocytes and DCs (Villani et al., 2017; Dutertre et al., 2019) using the myeloid atlas as the reference (Figures 2D and S2). An advantage of projecting both datasets to the same reference is that the subtypes can be evaluated against a broader set of reference cell types. Here, the Dutertre DC2 subsets aligned closely with atlas DC2 cells, while the spread of Villani clusters was much greater, and clearly aligning to respective DC and monocyte groups on the atlas. Annotation with a Capybara similarity estimate (Figure 2E) predicted three distinct DC2 (CD1c+) subsets were present, including an intermediate DC2 subset that sat between classical monocyte and DC2 (Figures 2E, 2F, and S2). The atlas data included blood samples taken from donors after vaccination (Banchereau et al., 2014) or cells isolated from inflammatory fluids (Segura et al., 2013) and these appear to represent distinct and reproducible activation phenotypes. Given the number of additional DC1 and DC2 subsets observed on the myeloid atlas using this approach, we suggest that there is greater heterogeneity in activated DC phenotypes than might be appreciated from individual studies.

We observed the new *AXL + SIGLEC6+* (AS-DC) subset that shared classical cell surface markers with pDCs and DC1s (Figure 2E). AS-DCs are the subset contaminating traditional pDC isolation strategies, responsible for observations that pDC can stimulate T cells (Villani et al., 2017). Projection of single-cell data like the Villani dataset onto the atlas highlights opportunities to delineate myeloid subtypes by combining the deep annotations associated with population data with the cellular resolution of single-cell approaches.

### Monocytes rapidly adapt to culture

**Figure 3:**
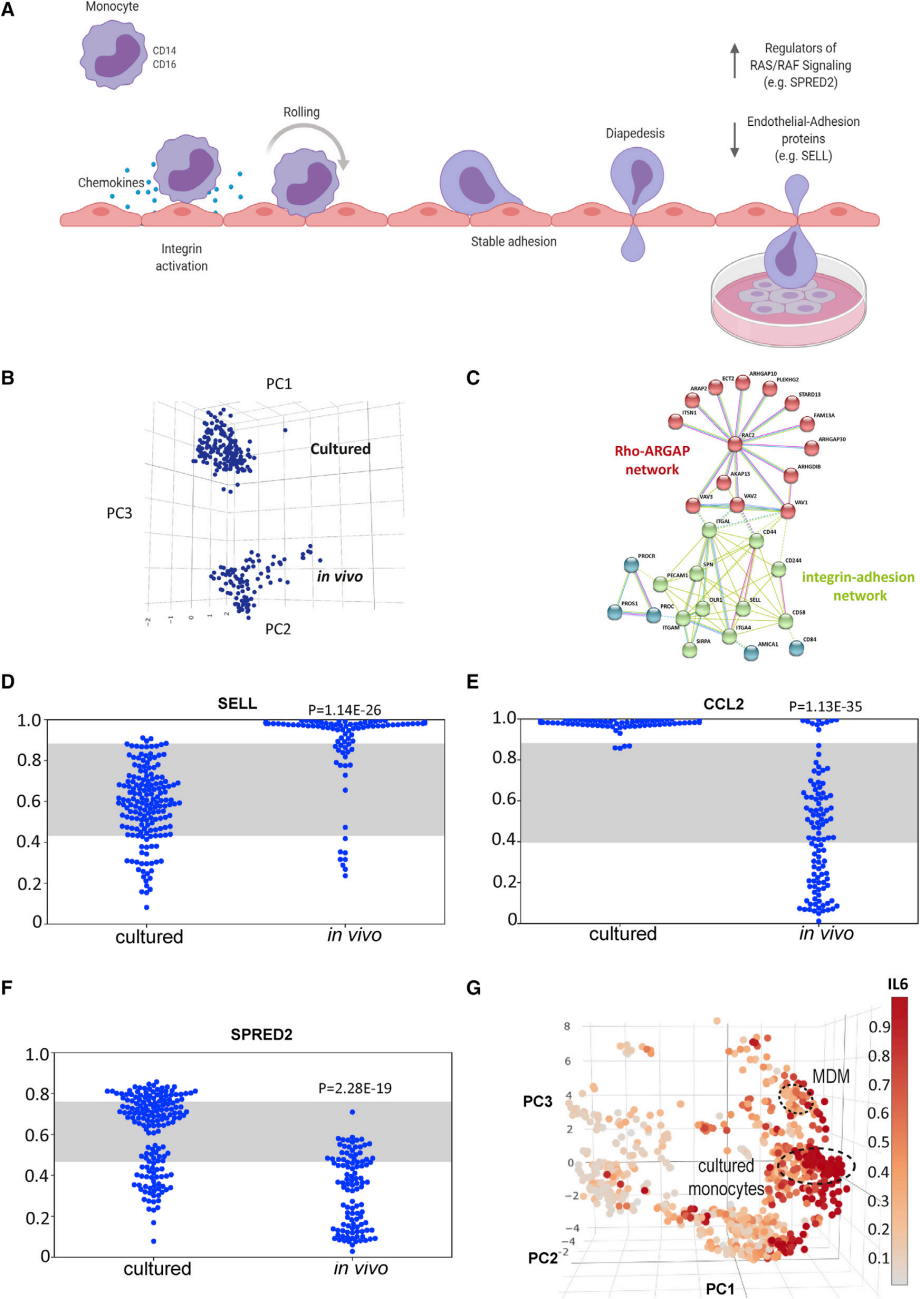
Monocytes acquire a culture phenotype (A) Schematic of rolling monocytes, highlighting cultured cells mimic many of the features of a monocyte after extravasation. (B) Cultured monocytes form a distinct cluster away from *in vivo* monocytes along PC3. (C) STRING-DB network of top-ranked genes differentially expressed between peripheral blood (*in vivo*, n = 107) and cultured monocytes (*ex vivo*, n = 171) indicating upregulation of cytoskeletal proteins and downregulation of endothelial-adhesion proteins. (D) Ranked expression (y axis) of gene involved in endothelial adhesion, *SELL*, comparing cultured monocytes (n = 171) with monocytes directly profiled from blood (*in vivo*, n = 107). Gray stripe indicates variance attributable to platform. p value: Mann-Whitney-Wilcoxon rank-sum test. (E) Ranked expression (y axis) of gene involved in the regulation of RAS/RAF signaling comparing cultured monocytes (n = 171) with monocytes directly profiled from blood (*in vivo*, n = 107). Gray stripe indicates variance attributable to platform. p value: Mann-Whitney-Wilcoxon rank-sum test. (F) Atlas colored by ranked expression of *IL6* (Scale bar: high ranked expression [dark red] to low ranked expression [gray] indicating axis of activation). Cultured (*ex vivo*) monocytes and MDMs highlighted. (G) Ranked expression (y axis) of chemokine *CCL2* comparing cultured monocytes (n = 171) with monocytes directly profiled from blood (*in vivo*, n = 107). Gray stripe indicates variance attributable to platform. p value: Mann-Whitney-Wilcoxon rank-sum test. See also Table S4.

Monocytes are post-mitotic blood cells derived from bone marrow that are short-lived in circulation and can repopulate macrophages in some tissue niches. The largest population of circulating monocytes is marked by high expression of the LPS co-receptor CD14, which is typically used to isolate monocytes from blood. Intermediate and nonclassical subsets are marked by acquisition of the type III FcRƔ, CD16 (Schmidl et al., 2014) and are included in the atlas. Cultured monocytes have been previously described as “activated,” but while we observe a distinct culture phenotype, the transcriptome of cultured cells mimics many of the features of a monocyte after extravasation into tissue (Figures 3A–3C). For example, cultured monocytes are typified by a decrease in endothelial-adhesion proteins associated with monocyte rolling prior to extravasation, including the selectin *SELL* (Figure 3D), and increased expression of *CCL2* (Figure 3E), associated with monocyte and macrophage migration into tissue. Regulators of RAS/RAF signaling including *SPRED2* (Wakioka et al., 2001) have an elevated expression in cultured monocytes (Figure 3F), consistent with spreading and migration across tissue culture plastics. *In vitro* differentiation of monocytes to MDMs typically requires several days of exposure to growth factors such as macrophage colony-stimulating factor (M-CSF; CSF-1) or granulocyte-macrophage colony-stimulating factor (GM-CSF). These group distinctly from the cultured monocyte cluster, spreading farther upward along the culture axis (Figure 3G).

The culture phenotype acquired by monocytes appears to be a prelude to activation, which can be observed along an adjacent axis in Figure 3F, and is exemplified by the expression of *IL6*. Pathogen-activated phenotypes of cultured monocytes are typified by high expression of this and other cytokines, but culture alone is not sufficient to induce cytokine expression. Culture induces the expression of *SLAMF1* (Table S4), which has shown to be necessary for TLR4 activation in human macrophages (Yurchenko et al., 2018), and cultured monocytes express higher levels of *ITGB8* than circulating monocytes (Table S4), a factor that is necessary for activating latent transforming growth factor (TGF)-β (Kelly et al., 2018).

### Pluripotent stem cell–derived macrophages do not recapitulate hematopoietic ontogenies

Many PSC-derived systems recapitulate fetal, rather than adult phenotypes, so it is no surprise that others have argued that PSC-derivation protocols mimic primitive rather than definitive myeloid biology. PSC-macrophages, including PSC-microglia and PSC-Kupffer cells, did form an extended group that was associated with high expression of the human homologue of the *F4/80* antigen, *ADGRE1*, as well as high expression of lipid-scavenging receptors such as *SCARB1* (Table S5).

**Figure 4:**
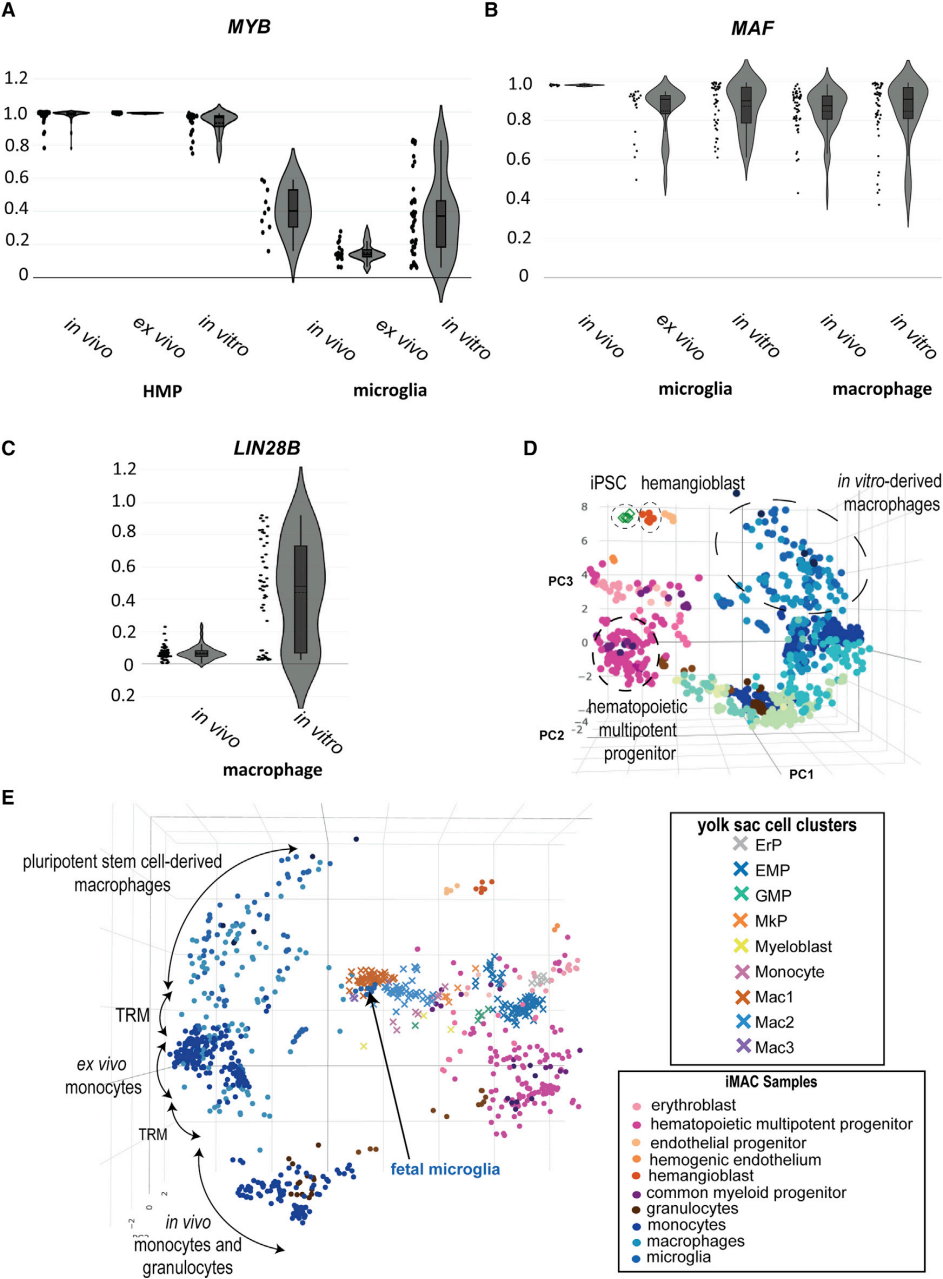
Pluripotent stem cell–derived macrophages do not recapitulate hematopoietic ontogenies (A) Violin plot comparing expression of *MYB* in *in vivo* (n = 91), *ex vivo* (n = 33), and *in vitro* (n = 77) hematopoietic multipotent progenitors (HMP) and *in vivo* (n = 10), *ex vivo* (n = 21), and *in vitro* (n = 43) microglia. In vitro–derived HMP were from pluripotent (n = 21) or HSC (n = 56) sources. *In vitro*–derived microglia were entirely sourced from PSC. Y-axis shows ranked expression. (B) Violin plot comparing expression of *MAF* in *in vivo* (n = 10), *ex vivo* (n = 21), and *in vitro*- (n = 43) derived microglia and *in vivo* (n = 51) and *in vitro* (n = 50) macrophages (including gut, synovial, macrophage). Y-axis shows ranked expression. (C) Violin plot comparing *LIN2*8B expression from *in vivo* (n = 51) and *in vitro*- (n = 50) derived macrophages (gut, synovial, macrophage). Y-axis shows ranked expression. (D) Atlas with samples colored by cell type with projection of iPSC samples highlighting their position in comparison to *in vitro*–derived macrophages, HMPs and hemangioblast. (E) Single-cell projection of (Bian et al., 2020) human fetal yolk sac cell clusters onto the atlas following an eight-cell aggregation. See also Figure S3 and Tables S5 and S6.

The ontogeny argument is largely based on *MYB* expression, which is associated with definitive hematopoiesis and has high expression in hematopoietic progenitor cells isolated from bone marrow. It is clear that *MYB* is not required for PSC-derived myelopoiesis, as macrophages can be grown from *MYB* knockout embryonic stem cells (Buchrieser et al., 2017). Nevertheless, *MYB* is highly and ubiquitously expressed in PSC-hematopoietic multipotent progenitors, including common myeloid progenitors and hemogenic endothelium (Figure 4A). *MYB* was expressed in primary microglia, downregulated by culture in primary microglia, but was nonetheless consistently detectable in most PSC-derived microglia, with expression highest in PSC-derived datasets cocultured with astrocytes or neurons (Pandya et al., 2017) or TGF-β withdrawal in the final stages of culture (Abud et al., 2017; Pandya et al., 2017). Given the dynamic behavior of *MYB* in cultured myeloid cells, we conclude that its expression is a poor indicator of ontogeny.

Some other phenotypes previously attributed to ontogeny in PSC-derived cells may rather reflect a more general culture context. For example, *ADGRE1* (*F4/80*) expression has been attributed to yolk sac–derived myeloid cells in mouse (Schulz et al., 2012). While high on PSC-derived cells, *ADGRE1* is also clearly upregulated in *ex vivo* cultured macrophages. This is exemplified by primary human microglia, which have low expression of *ADGRE1* in comparison with *ex vivo* culture or PSC-derived cells (Tables S5 and S6). In contrast, *MAF* expression is indistinguishable in macrophages of different origin (Figure 4B). *LIN2*8B expression was one of the more distinguishing markers of PSC-derived macrophages compared with *in vivo* cells (Figure 4C), which may point to incomplete silencing of the Let7 microRNA pathway. *LIN2*8B expression is characteristic of myeloid leukemias, and might be argued that its expression represents an immature proliferative state (Rowe et al., 2016; Zhou et al., 2017).

To further assess whether PSC-macrophages follow a fetal trajectory, we projected two external data types onto the atlas. The first were undifferentiated pluripotent stem cells, which together with the PSC-spread appear to form a differentiation arc that is orthogonal to the hematopoietic multipotent progenitor cells included in the original atlas (Figure 4D). We next projected primitive human hematopoietic progenitors from Carnegie staged embryos at CS11 to CS23 (Bian et al., 2020), which aligned closely to the definitive differentiation trajectory predicted in the original atlas (Figures 4E and S3). For example, we observed predominant erythroid overlap in early yolk sac progenitors, followed by rapid emergence of myeloid progenitors, and primitive macrophages in subsequent Carnegie stages. These do not overlap with the PSC-axis, but do clearly sit with similar late fetal microglia (20-week-old; Thion et al., 2018) and adult bone-marrow macrophage equivalents (Watkins et al., 2009). The fetal equivalence of PSC-macrophages or microglia is not supported by these data.

### Pluripotent stem cell–derived macrophages share features with tissue-resident macrophages despite poor maturation

Macrophages derived from human PSCs nonetheless do offer new opportunities to model *in vivo* macrophage biology. When reviewing the studies contributing to this atlas, we noted that PSC-derived macrophages are typically benchmarked against MDMs, or cultured primary cells, using a suite of phenotyping techniques. Each experiment includes a small number of samples for transcriptional profiling, with a few notable exceptions (Alasoo et al., 2018). We argue that, given the spectrum of possible resident tissue macrophage phenotypes, it would be more useful to compare PSC-derived cells against an atlas of possible macrophage phenotypes. While several groups reuse publicly available tissue macrophage data, the opportunity to carry out large-scale comparisons to different primary myeloid cells has been limited by the availability of relevant data on a compatible platform. By including macrophages from different tissues, we have shown that cultured tissue macrophages including *ex vivo* primary microglia or Kupffer cells shared a broad transcriptional signature with MDMs, and pluripotent stem cell–derived myeloid cells.

Microglia represent just over a third of PSC-directed myeloid differentiation studies in the atlas. Primary microglia included in the atlas include both *in vivo* isolated fetal and cultured *ex vivo* fetal and adult microglia. The profiles of *in vivo* isolated fetal microglia cluster apart from the spread of *ex vivo* cultured adult and fetal microglia (Figure S1B). These do not resolve into a unique cluster but share transcriptional phenotypes with PSC-derived and tissue-resident macrophages. There are exceptions that include some “cytokine-matured” PSC-derived microglia samples from (Abud et al., 2017). These are close to the *in vivo* fetal microglia samples of the PSC-microglia but are also closely associated with other primary tissue-resident macrophages from lung, joint, and gut. The atlas does provide an opportunity to review the expression of markers thought to distinguish microglia from other primary macrophages. *TMEM119*, for example, is largely restricted to primary or PSC-derived microglia, although some PSC-microglia samples have low expression of this marker (Figure S1C). *P2RY12* is variably expressed across all microglial samples, but its expression is also evident in different tissue-resident samples including those derived from gut and synovial tissues (Tables S5 and S6).

The majority of PSC*-*derived macrophages have low expression of HLA relevant genes including *CIITA*, a known master regulator of MHCII gene expression, which suggests poor maturation and limited capacity to present antigen to lymphocytes. Nevertheless, some *in vitro*–derived macrophages cultured with stimulating factors such as interferon-gamma or LPS (Figure S1D) do show inducible *CIITA* expression, demonstrating that they have the capacity to express antigen-presenting machinery. It is also worth noting additional culture conditions that result in high *CIITA* expression without interferon-stimulation (Figure S1D). This may be the result of long-term culture conditions for microglia samples (Abud et al., 2017), or reflect prior conditioning of myeloid progenitors (Honda-Ozaki et al., 2018).

### Pluripotent stem cell–derived macrophages display transcriptional hallmarks of efferocytosis

**Figure 5:**
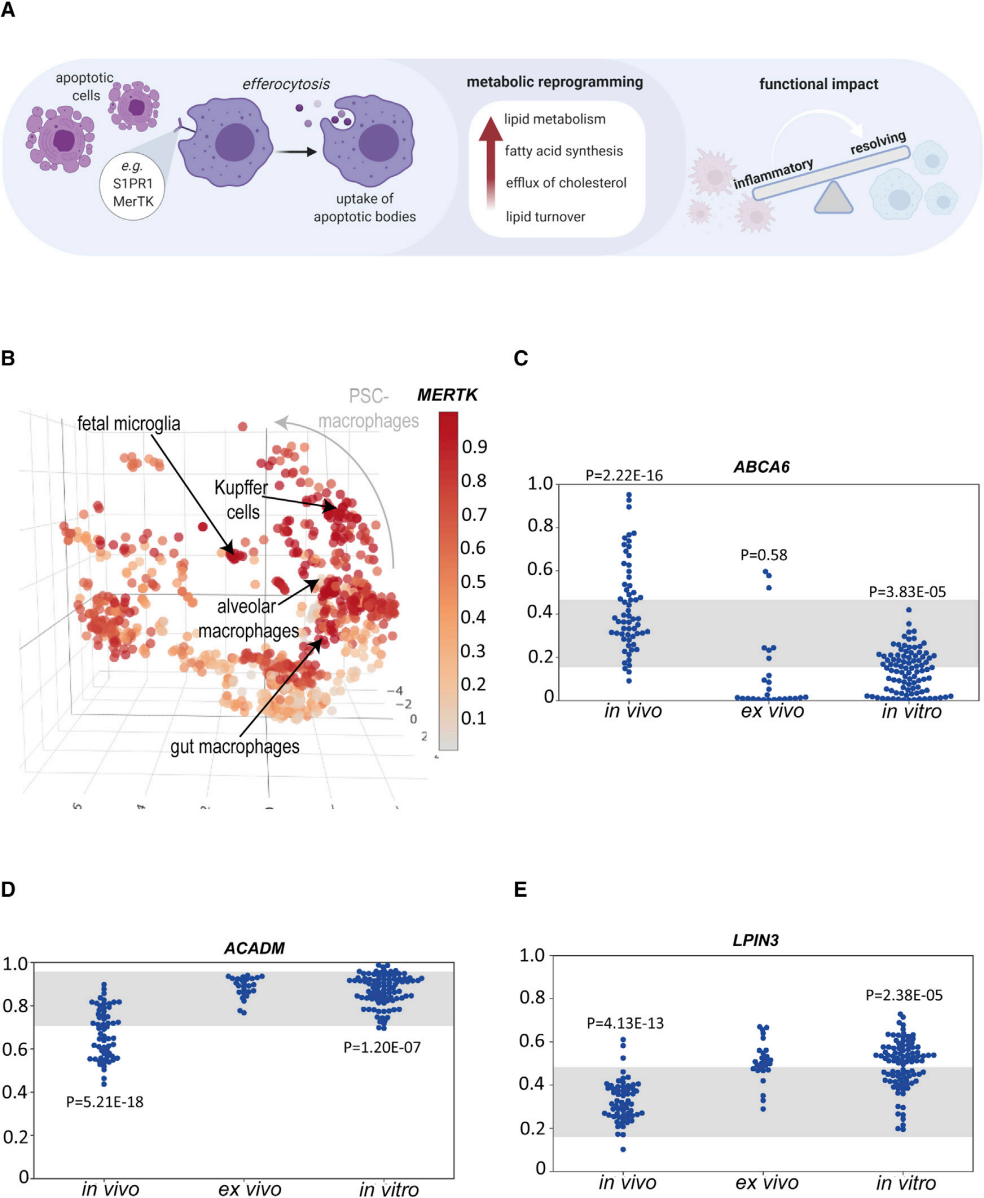
Pluripotent stem cell–derived macrophages display transcriptional hallmarks of efferocytosis (A) Schematic of impact of efferocytosis on cell metabolic reprogramming and function. (B) Atlas colored by ranked expression of MERTK (scale bar: high ranked expression [dark red] to low ranked expression [gray]. Positioning of fetal microglia, Kupffer cells, alveolar and gut macrophage samples highlighted. (C–E) Ranked expression (y axis) of genes comparing *in vivo* (n = 61), *ex vivo* (n = 26), and *in vitro*–derived (n = 96) macrophages (gut, synovial, Kupffer, microglia, macrophage) for (C) cholesterol efflux, (D) mitochondrial acyl-CoA dehydrogenase, and (E) phosphatidate phosphatase. p value: Mann-Whitney-Wilcoxon rank-sum test. See also Tables S5 and S6.

We observed expression of many of the hallmarks of efferocytosis in PSC-derived macrophages (Figure 5A). A recent study also demonstrated higher lipid uptake in PSC-macrophages compared with peripheral blood MDMs, concordant with higher expression of efferocytosis-related genes including *S1PR1* and *MERTK* (Cao et al., 2019). We confirm that *MERTK* is generally highly expressed in PSC-derived macrophages, but that there is also a tissue-resident distribution of *MERTK* expression, with very low levels observed in primary alveolar macrophages, and highest levels observed in human fetal microglia (Figure 5B). Efferocytosis, or apoptotic cell clearance, has broad immunomodulatory effects (reviewed by Elliott et al., 2017), and is known to modulate pro-inflammatory phenotypes and promote resolving qualities (Yamaguchi et al., 2014), consistent with the patterns of gene expression observed in cultured macrophages in the atlas. Active engulfment and clearance of cells by PSC-macrophages is clearly observed in the absence of any inflammatory activation (Video S1). Tissue-resident macrophages are known first-responders to tissue damage and are key in orchestrating inflammation and its subsequent resolution. This appears to be a phenotype that is selected for in cultured macrophages.

Video S1. Active engulfment and clearance of cells by pluripotent stem cell–derived macrophages

Lipid homeostasis is an important role for resident tissue macrophages. A high proportion of genes differentially expressed between *in vitro*–derived macrophages/microglia/Kupffer cells and tissue-resident cells are involved in lipid transport, catabolism and in buffering the cells from concomitant stresses associated with lipid turnover. For example, reduced expression of *ABCA6* is consistent with high efflux of cholesterol from these macrophages (Figure 5C). Higher levels of mitochondrial acyl-CoA dehydrogenase *ACADM* (Figure 5D) and phosphatidate phosphatase *LPIN3* (Figure 5E) are also consistent with high lipid turnover.

There has been growing interest in the importance of metabolic reprogramming in macrophage responses, so we asked whether media supplementation could explain the spread of PSC-derived macrophages on the atlas. All PSC-derivation protocols supplement media with fatty or amino acids, including L-Glutamine, nonessential amino acids (NEAA), linoleic and linolenic acids. Some methods add fetal bovine or calf serum, but there was no obvious correlation between serum addition and culture phenotype. Media factors are ubiquitous (Table S7) and no combination of supplement could explain the differences between PSC and cultured primary macrophages.

### Pluripotent stem cell–derived macrophages express high levels of collagen

**Figure 6:**
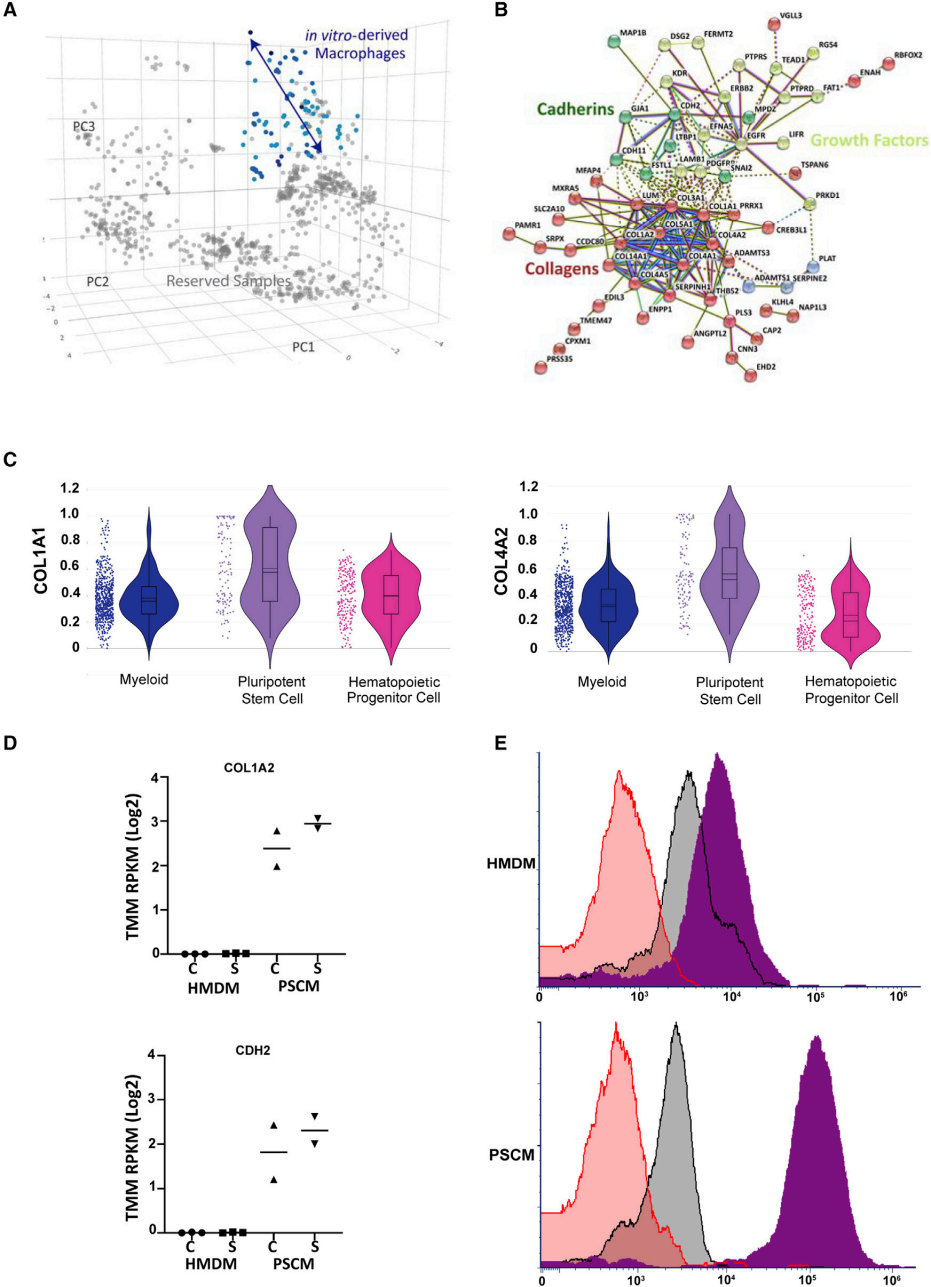
Pluripotent stem cell–derived macrophages express high levels of collagen (A) Atlas colored by cell source to highlight *in vitro*–derived macrophages used for regression testing. (B) STRING_DB Protein-Protein network of *in vitro*–derived macrophages highlights enrichment of collagen, growth factor and cadherin networks. Line color indicates the type of interaction evidence. Light blue solid lines indicate known interactions from curated databases, solid pink line indicates known interactions that have been experimentally determined, bright green lines indicate gene neighborhood predicted interactions, red lines indicate gene fusions predicted interactions, dark blue lines indicate gene co-occurrence predicted interactions, yellow/green lines indicate textmining, black lines indicate co-expression, and light purple lines indicate protein homology. (C) Violin plots of myeloid- (n = 584), pluripotent stem cell- (n = 116) and hematopoietic progenitor- (n = 201) derived cells comparing expression of collagen genes (*COL1A1* and *COL4A2*). Y-axis shows ranked expression. (D) mRNA-seq *COL1A2* and *CDH2* gene expression in human peripheral blood monocyte-derived macrophages (HMDM) (n = 3) and human pluripotent stem cell–derived macrophages (PSCM) (n = 2) samples (C = control, S = stimulated with 10 ng/mL lipopolysaccharide for 2 h). (E) Intracellular flow cytometry analysis of HMDM and human pluripotent stem cell–derived macrophages (PSCM), representative of two experimental repeats (n = 2 HMDM, n = 2 PSCM). Red = no primary antibody control, Black = isotype control, Purple = Type I Collagen stain. See also Tables S7 and S8.

We further examined the genes that are most correlated with PSC-derived macrophages and that distinguish PSC-macrophages from the tissue-resident populations. Gene set enrichment analysis (Figure 6A) revealed that the most significantly impacted pathways involved collagen production (Table S8). An STRING protein-protein interaction network (Figure 6B) shows that this phenotype is significantly enriched for highly connected matrix remodeling, collagen deposition and cadherin-mediated cell-cell and cell-matrix interactions. Initial observations on analysis of myeloid-, PSC-, and hematopoietic progenitor-derived cells, highlighted higher expression of collagen genes in PSC-derived cells (Figure 6C). Collagen production and deposition alongside extracellular matrix remodeling are processes involved in wound healing and scarring. Macrophages are instrumental in instructing tissue repair, particularly through the production of growth factors such as TGF-β, insulin growth factor 1, and platelet-derived growth factor (Shook et al., 2018). Secreted growth factors drive fibroblasts and endothelial cells to produce extracellular matrix components, promoting keloid formation as well as angiogenesis. This model has macrophages influencing collagen deposition by neighboring stromal cells; however, macrophages have been demonstrated to contribute to collagen deposition in mouse and zebrafish injury models (Simões et al., 2020).

Perhaps PSC*-*derived cells are being driven to adopt a pro-fibrotic phenotype because of their method of derivation; however, regression analysis of serum or TGF-β supplementation failed to reveal a strong correlation with the collagen network (Table S8). To further investigate whether this phenotype could be attributed to culture conditions, we cultured freshly isolated peripheral blood monocytes and human PSC-macrophage progenitors with the same culture media, including the presence of M-CSF and serum, for 5 days to drive macrophage differentiation. On day 5, cells were either stimulated with lipopolysaccharide for 2 h before extraction or extracted as control samples for sequencing analysis. The same enriched collagen and cadherin networks were observed in PSC-macrophages, but little to no expression of collagen was measured in the MDMs, regardless of whether cells were stimulated or not (Figure 6D). Intracellular flow cytometry analysis of type I collagen in PSC- and monocyte-derived cells (Figure 6E) showed that type I collagen proteins were only observable in PSC-macrophages. While culture media has a major impact on macrophage phenotype, it is not the main driver for the network of matrix proteins expressed by PSC-macrophages.

## Discussion

Human macrophage biology is integral to the development of homeostasis and disease mechanisms in every tissue in the body, but our understanding of human myeloid biology is limited by the quality by the models available to us. Here we describe an integrated myeloid transcriptome atlas as a resource to identify myeloid cells in single-cell datasets and to benchmark *in vitro* models of *in vivo* biology.

Transcriptional benchmarking of myeloid subsets, and particularly those benchmarking new derivation methods, typically draw on a small number of reference samples. Standard analysis workflows include normalization methods that remove technical batch effects and harmonize the behavior of samples assigned to the same class. When combining data that confounds technical batch with the biology of interest, this process inevitably overstates similarities within groups and overemphasizes differences between groups. This is an issue that is not restricted to bulk profiling methods but is also true of single-cell data integration methods, including popular methods such as Seurat and Harmony. Any normalization approach that rewards similarity between clusters and penalizes heterogeneity between datasets may introduce bias to subsequent analyses (see (Cahan et al., 2021; Tian et al., 2019). Data harmonization approaches are important to enumerate overlapping cell classes between disparate datasets and are complementary to approaches such as the variance filtering method described here. By providing a reference atlas constructed from a large number of well phenotyped and curated published data, we offer a readily accessible tool for benchmarking new models of myeloid biology. We demonstrate reproducible classification of major myeloid cell classes, including the influence of culture or derivation method on the phenotype of those cells. Projection of external data into the atlas further demonstrates that these phenotypic patterns are reproducible, even at the resolution of the single cell.

Our analyses highlight that there is room for improvement in the development of *in vitro* model systems that attempt to mimic *in vivo* counterparts. We demonstrate that cord blood–derived DCs differentiated *ex vivo* from monocytes or CD34 progenitors do not adequately capture key aspects of *in vivo* myeloid biology. Likewise, by benchmarking curated public data of PSC-macrophages and their precursor cells against the atlas, it is apparent that these represent neither definitive nor primitive myelopoiesis, or rather, that they imperfectly recapitulate aspects of both. PSC-conditions clearly do not mimic the developmental time frame or tissue niche of yolk sac, fetal liver, or bone marrow. PSC-macrophages do recapitulate many aspects of *ex vivo* cultured tissue macrophages, but there is little evidence for cultured microglia being distinct from other cultured macrophage models. PSC-macrophages display transcriptional hallmarks of efferocytosis and surprisingly collagen production, which may suggest that the derivation process preference is for a reparative phenotype, and more work is needed to ensure that these are not promoting a pro-fibrotic phenotype.

The atlas is implemented in Stemformatics.org and is available as an interactive 3D or 2D PCA graph that can be explored by sample type or gene expression, or both side-by-side. All data and code are publicly available. Users are able to select sample groups based on annotated categories of cell type, cell origin, disease, or activation status. Users may generate, and export, boxplots or violin plots of their selected gene, customizing how samples are grouped in gene summary plots. Atlas colors are fully customizable to assist users better distinguish variables of interest. The myeloid atlas enables users to upload their own gene expression data, including single-cell data, to benchmark cell types against the atlas for rapid and intuitive cell classification. The resource is scalable and will grow as the availability of new tissue-resident samples and myeloid models become available.

## Experimental procedures

Atlas construction was developed as described in (Angel et al., 2020) and is composed of 44 datasets, 901 samples, and 3,757 genes. Samples can be colored by cell type, sample source, progenitor type, tissue, disease, activation status, or dataset. Sample sources are classified as *in vivo* if directly profiled from tissue or blood, *ex vivo* if isolated and further cultured, or *in vitro* if differentiated in a dish. Full details are in the supplemental experimental procedures.

### Cell lines and ethics approvals

Stem cell work was carried out in accordance with The University of Melbourne ethics committee HREC (approval 1851831). Stem cell lines used were PB001.1 (Vlahos et al., 2019), a kind gift from the Stem Cell Core Facility at the Murdoch Children's Research Institute, and HDF51((Jones et al., 2013); RRID:CVCL_UF42) was kindly provided to ALL by Prof. Jeanne Loring (The Scripps Research Institute, San Diego, CA). Monocytes were isolated from buffy coat, which was obtained from the Australian Red Cross Blood Service in accordance with The University of Melbourne ethics committee HREC (approval 1646608).

### Data and code availability

Messenger RNA-seq data are available through accession GSE150893. All public accessions are listed in Table S6. Stemformatics code, atlas code and example datasets for projection onto the atlas are publicly available at https://github.com/wellslab/myeloid_atlas.

## Author contributions

Conception N.R., J.C., C.A.W.; Experimental Investigation and Interpretation N.R., V.J., J.G., C.A.W.; Experimental Resources A.L.L., C.A.W.; Methodology P.W.A., J.C., Y.D.; Data provider N.R., M.K.S., S.M.; Curation N.R., M.R., C.M.P., C.A.W.; Statistical analysis Y.D., K.A.L.C., P.W.A.; Writing – original draft N.R., C.A.W.; Writing - review and editing N.R., P.W.A., S.M., A.L.L., K.A.L.C., J.C., C.A.W.; Supervision C.A.W., A.L.L., K.A.C.; Project Funding – C.A.W.

## Conflict of interests

C.A.W. is an Associate Editor at *Stem Cell Reports*.

## Supplemental information

Document S1. Supplemental experimental procedures, Figures S1–S3, and Table S8

Table S1. Datasets and samples to compile atlas and single-cell projection, related to Figure 1 Tissue-resident macrophages and dendritic cells (DCs) from peripheral blood, spleen, thymus, joint, lung, gut, brain, and liver. Samples also included monocytes from peripheral and cord blood, as well as *in vitro* differentiated DCs from cord blood progenitors or monocyte-derived macrophages. Columns include dataset accession ID, platform, Stemformatics Dataset ID, number of samples, tier categorization, cell type and relevant tissue/organism part.

Table S2. Differential analysis of tissue-resident macrophages, related to Figure 1 Differential analysis of tissue-resident macrophages grouped into either colon/ascites-ovarian cancer (n = 19), lung/synovium (n = 31), and blood/liver/brain (n = 49) based on distribution in the atlas. Columns refer to gene symbols, p values recalculated by Mann-Whitney-Wilcoxon rank-sum test, mean and standard deviation.

Table S3. *In vivo* vs. *ex vivo* vs. *in vitro* dendritic cells, related to Figure 2 Comparison of gene expression of *in vivo* (n = 57), *ex vivo* (n = 105), and *in vitro*- (n = 57) derived dendritic cells. Columns refer to gene symbols, p values recalculated by Mann-Whitney-Wilcoxon rank-sum test, mean and standard deviation.

Table S4. *In vivo* vs. *ex vivo* monocytes, related to Figure 3 Comparison of gene expression of *ex vivo* (n = 171) and *in vivo* (n = 107) monocytes. Columns refer to gene symbols, p values recalculated by Mann-Whitney-Wilcoxon rank-sum test, mean and standard deviation.

Table S5. *In vivo* vs. *ex vivo* vs. *in vitro* macrophages, related to Figures 4 and 5 Comparison of gene expression of *in vivo* (n = 61), *ex vivo* (n = 26), *in vitro*- (n = 96) derived macrophages (gut, synovial, Kupffer, microglia, macrophage). Columns refer to gene symbols, p values recalculated by Mann-Whitney-Wilcoxon rank-sum test, mean and standard deviation.

Table S6. *In vivo* vs. *ex vivo* vs. *in vitro* microglia, related to Figures 4 and 5 Comparison of gene expression of *in vivo* (n = 10), *ex vivo* (n = 21), and in vitro- (n = 43) derived microglia. Columns refer to gene symbols, p values recalculated by Mann-Whitney-Wilcoxon rank-sum test, mean and standard deviation.

Table S7. A review of factors added during pluripotent stem cell differentiation into monocytes/macrophages/microglia/Kupffer cells, related to Figure 6 Comparison of differentiation strategies that have been published describing the derivation of macrophages, microglia, Kupffer cells, and monocytes from pluripotent stem cells.

Document S2. Article plus supplemental information
