# Supplementary material for: An integrated analysis of human myeloid cells identifies gaps in in vitro models of in vivo biology
Source: Stem Cell Reports. 2021 May 13;16(6):1629–43. doi: 10.1016/j.stemcr.2021.04.010 (PMC8190595; doi:10.1016/j.stemcr.2021.04.010)
Supplement: Document S1. Supplemental experimental procedures, Figures S1–S3, and Table S8 [file mmc1.pdf]

Stem Cell Reports, Volume *16*

Supplemental Information

An integrated analysis of human myeloid cells identifies gaps in *in vitro* models of *in vivo* biology

Nadia Rajab, Paul W. Angel, Yidi Deng, Jennifer Gu, Vanta Jameson, Mariola Kurowska-Stolarska, Simon Milling, Chris M. Pacheco, Matt Rutar, Andrew L. Laslett, Kim-Anh Lê Cao, Jarny Choi, and Christine A. Wells

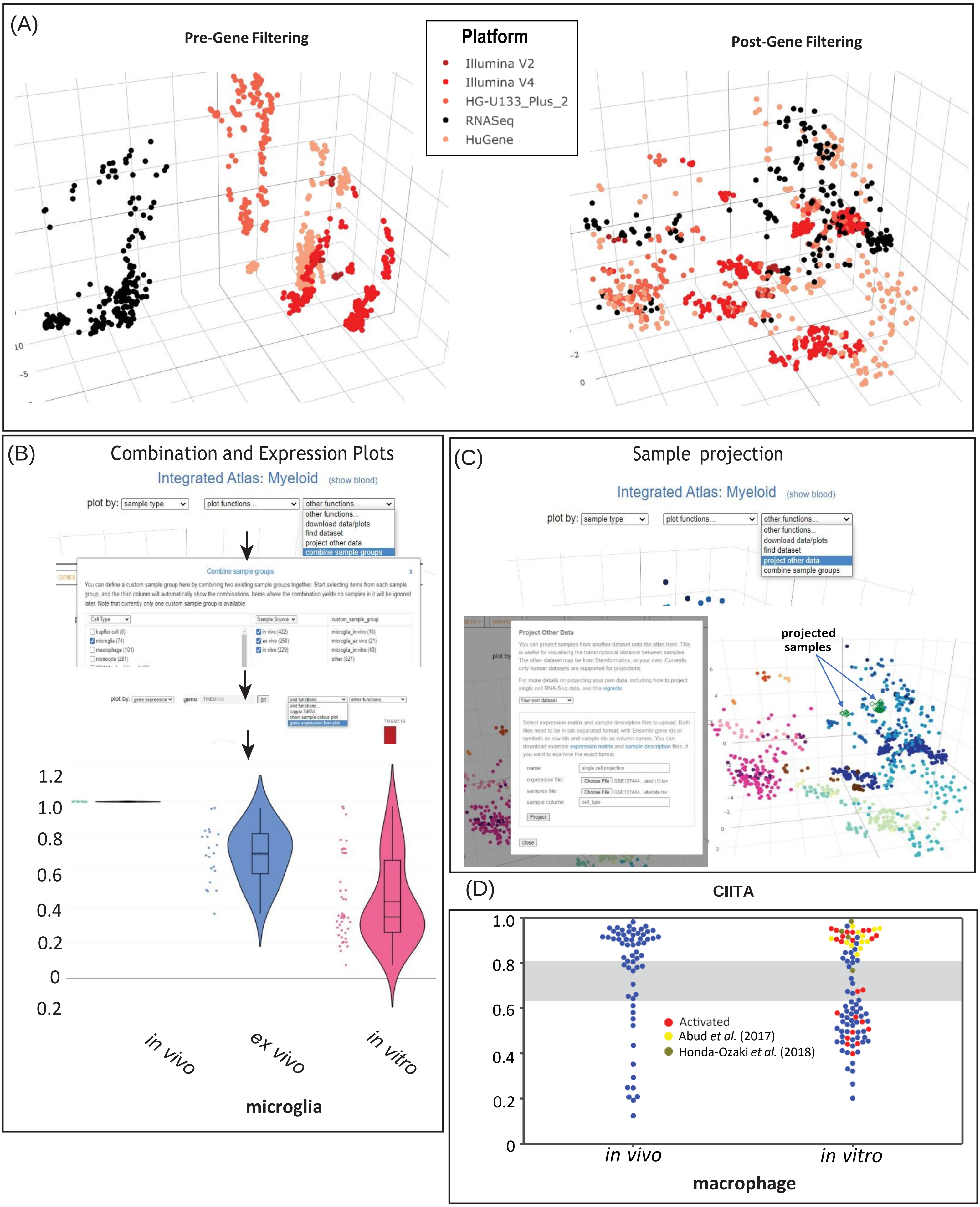

Figure S1: A reference atlas and resource for human myeloid biology. Related to Figure 1.

(A) Pre-gene filtering (left) and Post-gene filtering (right) atlas coloured by platform: red various microarray platforms, black RNAseq platforms. (B) Sample group combination and generation of expression plot example. Violin plot showing ranked gene expression for *TMEM119* of *in vivo*, *ex vivo* and *in vitro* microglia (C) Outline of sample projection processing of (Mancuso et al., 2019) dataset samples of Pluripotent Stem Cell-microglia samples (D) Ranked expression (Y-axis) of Class II transactivator (CIITA) *in vivo* versus *in vitro*-derived macrophages (gut, synovial, kupffer, microglia, macrophage). Red –activated, yellow – (Abud et al., 2017) microglia samples, khaki- (Honda-Ozaki et al., 2018) macrophage samples.

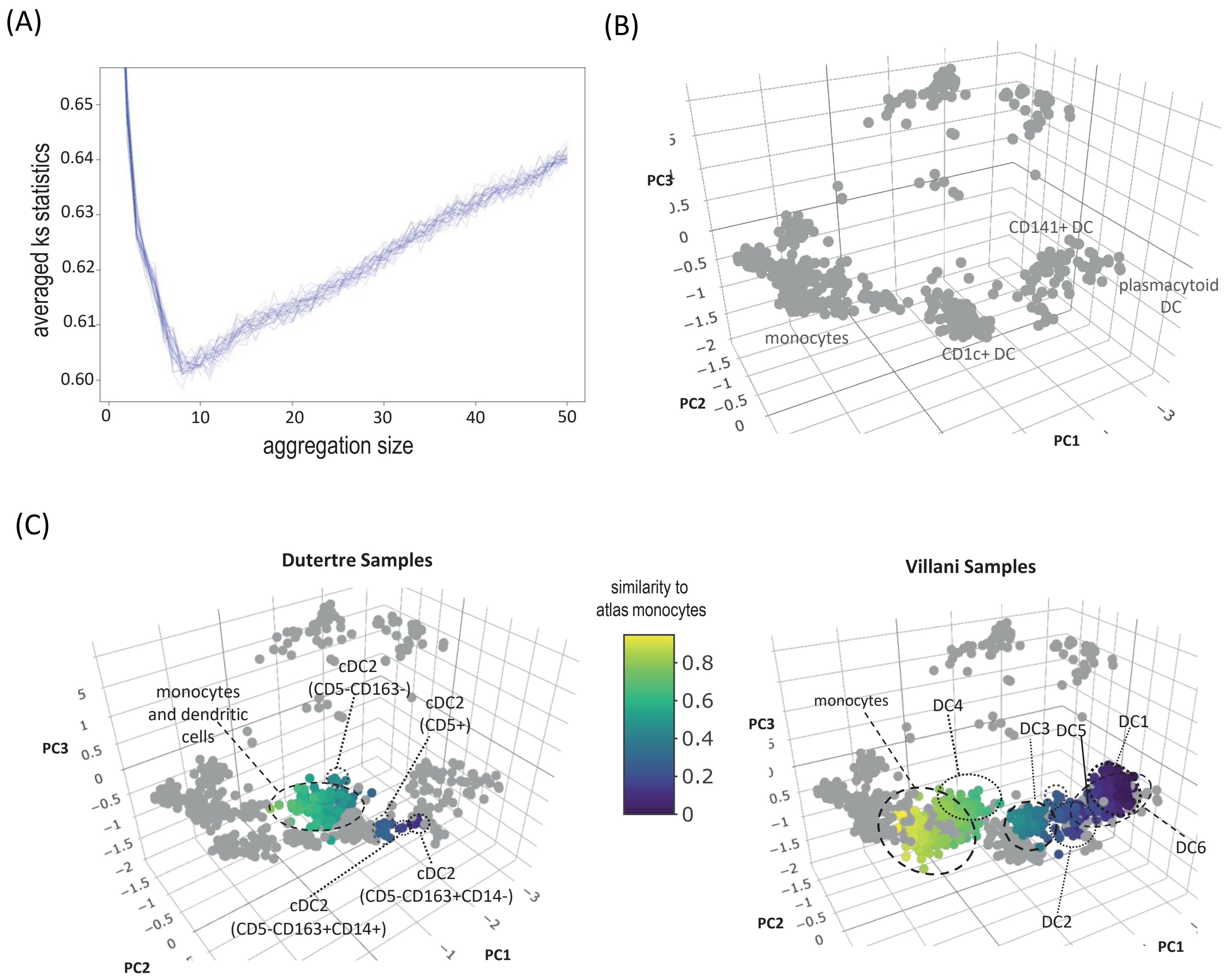

Figure S2: Single cell aggregation and projection. Related to Figure 2.

(A) Kolmogorov–Smirnov (KS) statistics (y-axis) to assess the difference in gene expression distribution between pseudo-bulk single cells DC6 and bulk sample plasmacytoid dendritic cells from the atlas, with respect to the number of single cells that are aggregated (x-axis). Each line indicates one of thirty random sub-samplings with replacement trial. KS statistics are calculated on each gene and averaged across all genes. A minimum KS statistic is obtained when aggregating 8 cells. (B) Atlas cell types before single cell projection (C) Single cell projection of (Dutertre et al., 2019) and (Villani et al., 2017) samples onto the atlas where 8 cells are aggregated based on (A).

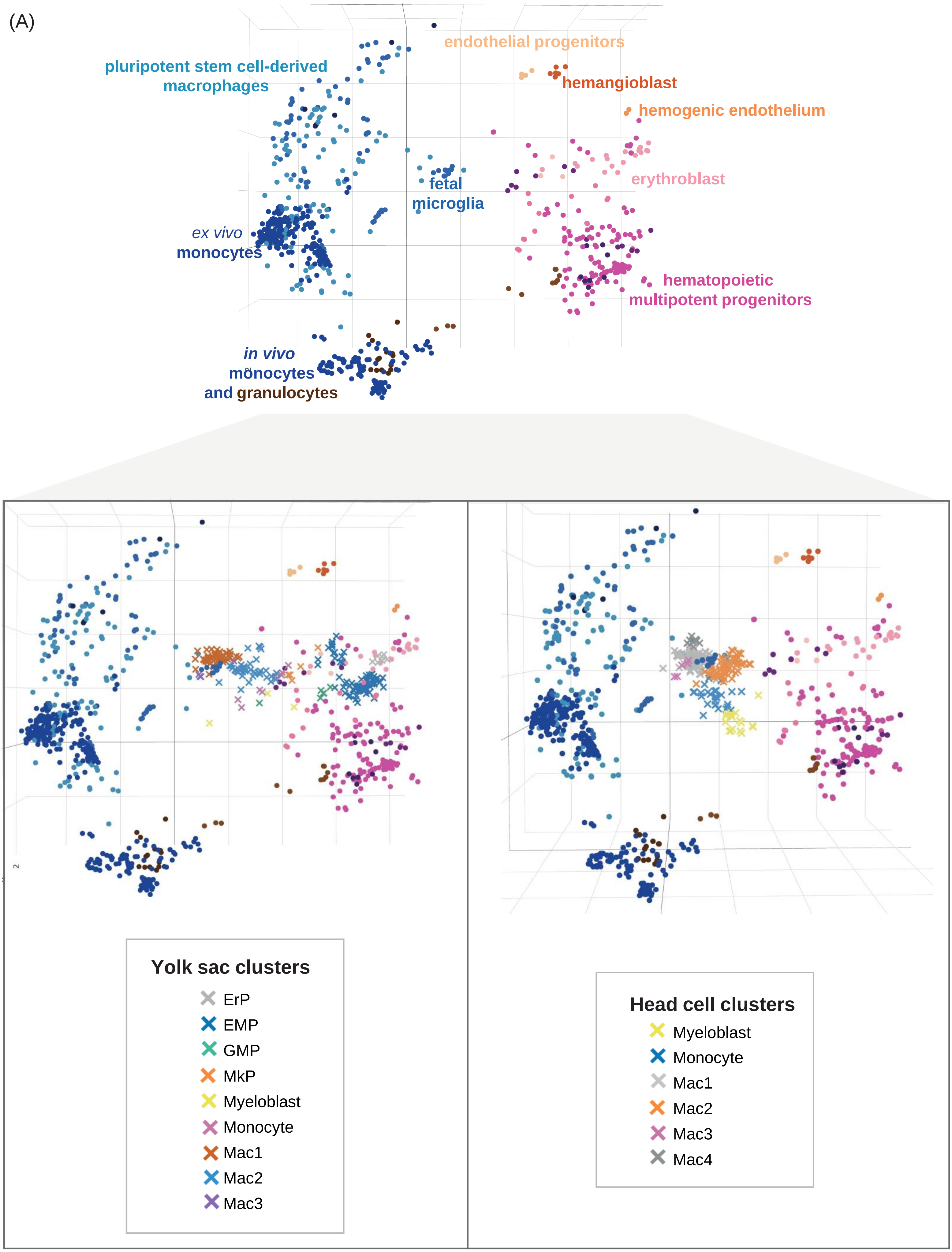

Figure S3: Fetal Ontogeny. Related to Figure 4.

Atlas coloured by cell type with (Bian et al., 2020) projection of single cell data from human fetal yolk sac and head.

Supplemental Tables

Table S1: Datasets and samples to compile atlas and single cell projection. Related to Figure 1.
Tissue resident macrophages and dendritic cells from peripheral blood, spleen, thymus, joint, lung, gut, brain and liver. Samples also included monocytes from peripheral and cord blood, as well as *in vitro* differentiated DCs from cord blood progenitors or monocyte-derived macrophages. Columns include dataset accession ID, platform, Stemformatics Dataset ID, number of samples, tier categorization, cell type and relevant tissue/organism part.

Table S2: Differential analysis of tissue-resident macrophages. Related to Figure 1.
Differential analysis of tissue-resident macrophages grouped into either colon/ascites-ovarian cancer (n=19), lung/synovium (n=31) and blood/liver/brain (n=49) based on distribution in the atlas. Columns refer to gene symbols, P-values recalculated by Mann-Whitney-Wilcoxon rank-sum test, mean and standard deviation.

Table S3: *in vivo* vs. *ex vivo* vs. i*n vitro* dendritic cells. Related to Figure 2.
Comparison of gene expression of *in vivo* (n=57), *ex vivo* (n=105) and *in vitro*- (n=57) derived dendritic cells. Columns refer to gene symbols, P-values re-calculated by Mann-Whitney-Wilcoxon rank-sum test, mean and standard deviation.

Table S4: *in vivo* vs. *ex vivo* monocytes. Related to Figure 3.
Comparison of gene expression of *ex vivo* (n=171) and *in vivo* (n=107) monocytes. Columns refer to gene symbols, P-values re-calculated by Mann-Whitney-Wilcoxon rank-sum test, mean and standard deviation.

Table S5: *in vivo* vs. *ex vivo* vs. *in vitro* macrophages. Related to Figure 4 and Figure 5.
Comparison of gene expression of *in vivo* (n=61), *ex vivo* (n=26), *in vitro*- (n=96) derived macrophages (gut, synovial, kupffer, microglia, macrophage). Columns refer to gene symbols, P-values recalculated by Mann-Whitney-Wilcoxon rank-sum test, mean and standard deviation.

Table S6: *in vivo* vs. *ex vivo* vs. *in vitro* microglia. Related to Figure 4 and Figure 5.
Comparison of gene expression of in vivo (n=10), ex vivo (n=21) and in vitro- (n=43) derived microglia. Columns refer to gene symbols, P-values re-calculated by Mann-Whitney-Wilcoxon rank-sum test, mean and standard deviation.

Table S7: A review of factors added during pluripotent stem cell differentiation into monocytes/macrophages/microglia/kupffer cells. Related to Figure 6.
Comparison of differentiation strategies that have been published describing the derivation of macrophages, microglia, kupffer cells and monocytes from pluripotent stem cells.

Table S8: Gene-Set Enrichment Analysis. Related to Figure 6.
Table of the top 10 Reactome pathways enriched in genes highly correlated with *in vitro*-derived macrophage spread. Enrichment: number of genes in the list/number of genes in that pathway (False Discovery Rate-value). Genes: multiple entries assigned to the same gene indicated by underlining of gene symbol with UniProt accessions in brackets.

Supplemental Video

Active engulfment and clearance of cells by pluripotent stem cell-derived macrophages.

Table S8: Gene-Set Enrichment Analysis

Table 1		
**Pathway**	**Enrichment**	**Genes**
Collagen biosynthesis and modifying enzymes	11/76 (3.76e-09)	ADAMTS3, COL1A2, COL4A2, SERPINH1, COL14A1, COL3A1, COL4A5, COL1A1, COL4A1 *(P02462, Q03692)*, COL5A1
Extracellular matrix organization	18/329 (4.48e-09)	ADAMTS1, COL1A1, COL4A1*(P02462, Q03692)*, COL5A1, LTBP1, PTPRS, ADAMTS3, COL1A2, COL4A2, KDR, LUM, SERPINH1, COL14A1, COL3A1, COL4A5, LAMB1, MFAP4
Collagen chain trimerization	9/44 (4.81e09)	COL14A1, COL3A1, COL4A5, COL1A1, COL4A1*(P02462, Q03692)*, COL5A1, COL1A2, COL4A2
Collagen formation	11/104 (2.39e-08)	ADAMTS3, COL1A2, COL4A2, SERPINH1, COL14A1, COL3A1, COL4A5, COL1A1, COL4A1*(P02462, Q03692)*, COL5A1
ECM proteoglycans	10/79 (2.39e-08)	COL1A1, COL4A1, COL5A1, PTPRS, COL1A2, COL4A2, LAMB1, COL3A1, COL4A5, LUM
Non-integrin membrane-ECM interactions	9/61 (3.83e-08)	COL1A1, COL4A1*(P02462, Q03692)*, COL5A1, COL1A2, COL4A2, LAMB1, COL3A1, COL4A5
Integrin cell surface interactions	10/86 (3.83e-08)	COL1A1, COL4A1*(P02462, Q03692)*, COL5A1, COL1A2, COL4A2, KDR, COL3A1, COL4A5, LUM
Assembly of collagen fibrils and other multimeric structures	9/67 (6.95e-08)	COL14A1, COL3A1, COL4A5, COL1A1, COL4A1*(P02462, Q03692)*, COL5A1, COL1A2, COL4A2
Collagen degradation	9/69 (7.93e-08)	COL14A1, COL3A1, COL4A5, COL1A1, COL4A1*(P02462, Q03692)*, COL5A1, COL1A2, COL4A2
Degradation of the extracellular matrix	11/148 (3.73e-07)	ADAMTS1, COL1A2, COL4A2, LAMB1, COL14A1, COL3A1, COL4A5, COL1A1, COL4A1*(P02462, Q03692)*, COL5A1

Table of the top 10 Reactome pathways enriched in genes highly correlated with *in vitro*-derived macrophage spread. Enrichment: number of genes in the list/number of genes in that pathway (False Discovery Rate-value). Genes: multiple entries assigned to the same gene indicated by underlining of gene symbol with UniProt accessions in brackets.

Supplementary Methods

Atlas construction:

Mapping, and analysis of microarray and RNA sequencing datasets were conducted in the Stemformatics platform. Scripts are available for download from the Stemformatics BitBucket (Choi et al., 2019)). All datasets and relevant samples (Supplementary Table 1) passed stringent quality control checks required for hosting on the Stemformatics platform. Quality control checks include evaluation of library quality, and inclusion of replicates associated with experimental design. These datasets were either already hosted on Stemformatics or were downloaded from public depositories and processed through the Stemformatics pipeline for inclusion.

Platform Effect Analysis and Gene Selection for PCA

This method assesses each gene independently to assess whether their expressions are affected by the experimental platform across all datasets, as described in detail in (Angel et al., 2020). Briefly, the initial step is to transform expression values from RNA Sequencing and Microarray into percentile values. The second step uses a linear mixed model to partition each gene's variance composition into three distinct categories: Variance explained by samples' platform sources, cell types and unexplained by the model. The threshold for gene selection was empirically determined based on a platform variance ratio of 0.2 to remove genes with platform effect for the first three principal components. A total of 3757 genes with low proportion of variance attributed to the platform effects relative to their total variance were then kept for PCA, thus ensuring that our atlas include a low platform dependency.

Quantification and Statistical Analysis

P-values were re-calculated using the Mann-Whitney-Wilcoxon rank-sum test (two-sided). This was implemented via the python (version 3.7.5) SciPy package version 1.3.1 (Virtanen et al., 2019). Multiple testing over the set of genes was accounted for with Bonferroni correction implemented in the statsmodels package (Seabold and Perktold, 2010)

Pseudo-bulk samples from Villani's Single Cell Data

Single cells were aggregated to form pseudo-bulk samples to mitigate library size differences between single cell and bulk data, and to project samples onto the atlas. Each group of (known) cell type in the single cell data was randomly sampled for k single cells with replacement. Aggregation consisted in summing up their expression profiles. k was determined by the number of sampling (i.e. how many pseud-bulk samples for each cell type) as half of the cell type's population size. The optimum aggregation size k was investigated by evaluating the similarity in distribution between the atlas' plasmacytoid dendritic cells and the aggregated pseudo-bulk DC6 samples using Kolmogorov–Smirnov (KS) statistic D averaged across all genes. KS statistic measured the difference between the empirical cumulative distribution functions of two groups of samples; the smaller the value, the closer the aggregated DC6 resembled the reference transcriptional profiles from the pDC in the bulk atlas. A minimum D value was obtained fo$k = 8$ across 30 iterations (Figure S2). Similar results were obtained for other cell types. Thus, every pseudo-bulk samples were aggregated from 8 single cells.

Capybara Cell Score

Capybara (Kong et al., 2020)cell scores was used to measure cell identities continuum of the pseudo-bulk samples using the atlas as the reference. Capybara cell scores were calculated by performing restricted linear regression of reference samples on each of the pseudo-bulk samples' expression profiles. Denote y_i the expression profile of thei^{th} pseudo-bulk sample of length G, where G represents the total number of genes in the data, and$X$ a $(G \times T)$ the reference matrix, where T represents the number of known cell types of interest. We considered 5 cell types: Dendritic cell, Monocyte, CD141+ dendritic cell, CD1c+ dendritic cell and Plasmacytoid dendritic cells. X is obtained by averaging the expression profiles of the Stemformatics myeloid samples according to their cell types. Capybara solves the optimization problem

$$argmin_{\beta} \quad (y_i - X\beta_i)^T(y_i - X\beta_i)$$

under the constraint that

$$\beta_{it} > 0 \quad \forall t \in \{1, 2, \ldots, T\}, \ \sum_{t}^{T} \beta_{it} < 1$$

where β_{it} is a regression coefficient, or cell score, for each pseudo-bulk sample i and each atlas cell type t. The cell score is obtained using quadratic programming implemented with R (version 3.6.2) package *quadprog version 1.5-8*(Turlach and Weingessel, 2019)).

Enrichment analysis and Protein-Protein Network

An enrichment analysis was conducted on the top 92 genes ranked by Pearson correlation (≥0.7) along the upward axis including in vitro-derived cells. Enriched pathways were identified using these genes at Reactome (Fabregat et al., 2018) and significance ranked by p-value/false discovery rate. A protein-protein network was generated using the top 92 genes on STRING-DB (Szklarczyk et al., 2019). Disconnected nodes not shown.

Pluripotent Stem Cell Differentiation

Human pluripotent stem cells were differentiated into macrophages based on protocol described by (Joshi et al., 2019; Ng et al., 2008; Yanagimachi et al., 2013) with modifications. Modifications were as follows: embryoid bodies were kept in rotational cultures without transference to matrigel plates for adherence, and the collection of progenitors from week 2 were immediately re-suspended in RPMI-1640 containing L-Glutamine (Life Technologies), 10% Fetal Bovine Serum and 100ng/mL CSF1 for macrophage differentiation (see macrophage differentiation).

Monocyte isolation

The blood was diluted with PBS at a 1:3 dilution and underlayed with Ficoll-Hypaque. The underlayed blood samples were centrifuged at 350g for 30 minutes at 24°C with no brake. Peripheral blood mononuclear cells were isolated from the interphase and washed twice by using MACs buffer (DPBS, 0.5% heat inactivated Fetal Bovine Serum, 2mM EDTA) and centrifuging at 400g for 5 minutes at 4°C. Cells were centrifuged at 400g for 5 minutes at 4°C and resuspended in 40µl MACs buffer per 107 cells. Monocytes were positively selected by a magnetic field using Human CD14 MicroBeads (MACS Miltenyi Biotec) and LS Columns (Miltenybiotec). These cells were plated for macrophage differentiation (see Macrophage differentiation)

Macrophage differentiation

Monocytes/progenitor cells were cultured in tissue-culture treated 6 well plates. Cells were cultured in RPMI-1640 medium containing L-Glutamine (Life Technologies) with 10% Fetal Bovine Serum and 100ng/ml recombinant Human M-CSF (R&D Systems; 216-MC) for 5 days. Media changes were carried out on day 4.

Flow Cytometry

HMDM and PSCM were collected and centrifuged at 400g for 5 minutes. Supernatant was aspirated and 5µl mouse serum was added to 'dry' pellets for 5 minutes on ice. Cells were resuspended in FACS Buffer (Hanks Balanced Salt Solution, 0.5% Human Serum Albumin) and stained with CD14 or matched isotype control antibodies on ice for 20 minutes then washed twice (3ml FACS Buffer, spun at 400g, 5 minutes). Resuspended cells were fixed with 4% paraformaldehyde (PFA) for 15 minutes at room temperature. PFA was washed out and cells rinsed twice in FACS buffer before resuspending in PBS and stored overnight at 4°C. Fixed cells were permeabilized with 0.1% Triton X-100 (in 1XPBS) for 10 minutes at room temperature and washed twice. Blocking buffer (0.3M glycine buffer, 10% Goat Serum, 1XPBS) was added to 'dry' pellets on ice for 1 hour. Cells were stained with antibodies to Type I Collagen or matched isotype control on ice for 20 minutes. Cells were washed twice then incubated with secondary antibody for 20 minutes on ice in the dark. Cells were then washed twice and resuspended for analysis.

Analysis as conducted on a CytoFLEX S flow cytometer (Beckman Coulter, Brea, CA) using CytExpert acquisition software. Post-acquisition analysis was performed with FCS Express 7 flow cytometry software.

**Flow Cytometry antibodies**	
CD14	Brilliant Violet 421™ anti-Human CD14 (BioLegend: Cat. No. 325628; RRID:AB_2563296)
CD14 Isotype	Brilliant Violet 421™ Mouse IgG1 (BioLegend: Cat. No. 400157; RRID:AB_10897939)
Type I Collagen Primary antibody	Rabbit Anti-Collagen I antibody (Abcam: Cat. No. ab264074)
Secondary Antibody	Goat Anti-Rabbit IgG H&L (Alexa Fluor®488) (Abcam: Cat. No. ab150077; RRID:AB_2630356)
Type I Collagen Isotype	Rabbit IgG monoclonal Isotype Control (Abcam: Cat. No. Ab172730; RRID:AB_2687931)

Stimulation Assay

On day 5 of differentiation, one well containing peripheral blood monocyte- or human pluripotent stem cell-derived macrophages were stimulated with 10ng/ml Lipopolysaccharide (LPS) (Sigma-Aldrich; *Salmonella enterica* serotype minnesota) for 2 hours. After stimulation period, media was aspirated, and the wells were washed twice with PBS (Ca2+Mg2+ free). before cell lysis using 2-mercaptoethanol (Sigma-Aldrich) and RNeasy Plus Lysis Buffer (Qiagen). Samples were placed into Eppendorf's and stored at -80°C before RNA extraction.

RNA extraction

Total RNA was isolated using the RNeasy® Plus Mini Kit (Qiagen) according to manufacturer's instructions. In summary: for the removal of genomic DNA, samples were placed into gDNA columns and centrifuged for 30 seconds at 8000g. Ethanol (70%) was mixed with the flow through and samples were transferred to RNeasy spin columns. The columns were centrifuged for 15 seconds at 8000g. Buffer RW1 was then added to the columns and columns were centrifuged for 15 seconds at 8000g. Buffer RPE was added to the columns and columns were centrifuged for 15 seconds. Buffer RPE was again added to the columns with centrifugation at 8000g for 2 minutes. Columns were then placed into new collection tubes and centrifuged at full speed for 1 minute to dry the membrane. RNase-free water was then added directly onto the column membrane and columns placed into Eppendorf's and centrifuged at 8000g for 1 minute to collect RNA. RNA quality and quantity were determined using a Tapestation (Agilent Technologies 2200). Samples were stored at -80°C. Zymo Research RNA Clean & Concentrator-25 Kit was used to pool replicates (from the same donor) together and elute in elute into smaller volume with maximum concentration.

RNA sequencing

RNA samples were processed by the Ramaciotti Centre for Genomics (University of New South Wales; Sydney). Illumina Novaseq_6000 was used for mRNA-sequencing.

Graphing software, Illustrations and video

Graphs for mRNA-seq gene expression were generated using Graphpad Prism. Violin plots were generated through the www.stemformatics.org platform. Schematic Figure illustrations were created with BioRender.com. Culture video of pluripotent stem cell-macrophages was captured using Lonza CytoSMART 2 system.

Supplementary References

Abud, E.M., Ramirez, R.N., Martinez, E.S., Healy, L.M., Nguyen, C.H.H., Newman, S.A., Yeromin, A. V., Scarfone, V.M., Marsh, S.E., Fimbres, C., et al. (2017). iPSC-Derived Human Microglia-like Cells to Study Neurological Diseases. Neuron *94*, 278-293.e9.

Angel, P.W., Rajab, N., Deng, Y., Pacheco, C.M., Chen, T., Lê Cao, K.-A., Choi, J., and Wells, C.A. (2020). A simple, scalable approach to building a cross-platform transcriptome atlas. PLoS Comput. Biol. *16*.

Bian, Z., Gong, Y., Huang, T., Lee, C.Z.W., Bian, L., Bai, Z., Shi, H., Zeng, Y., Liu, C., He, J., et al. (2020). Deciphering human macrophage development at single-cell resolution. Nature 1–6.

Choi, J., Pacheco, C.M., Mosbergen, R., Korn, O., Chen, T., Nagpal, I., Englart, S., Angel, P.W., and Wells, C.A. (2019). Stemformatics: visualize and download curated stem cell data. Nucleic Acids Res. *47*, D841–D846.

Dutertre, C.A., Becht, E., Irac, S.E., Khalilnezhad, A., Narang, V., Khalilnezhad, S., Ng, P.Y., van den Hoogen, L.L., Leong, J.Y., Lee, B., et al. (2019). Single-Cell Analysis of Human Mononuclear Phagocytes Reveals Subset-Defining Markers and Identifies Circulating Inflammatory Dendritic Cells. Immunity *51*, 573-589.e8.

Fabregat, A., Jupe, S., Matthews, L., Sidiropoulos, K., Gillespie, M., Garapati, P., Haw, R., Jassal, B., Korninger, F., May, B., et al. (2018). The Reactome Pathway Knowledgebase. Nucleic Acids Res. *46*, D649–D655.

Honda-Ozaki, F., Terashima, M., Niwa, A., Saiki, N., Kawasaki, Y., Ito, H., Hotta, A., Nagahashi, A., Igura, K., Asaka, I., et al. (2018). Pluripotent Stem Cell Model of Nakajo-Nishimura Syndrome Untangles Proinflammatory Pathways Mediated by Oxidative Stress. Stem Cell Reports *10*, 1835–1850.

Joshi, K., Elso, C., Motazedian, A., Labonne, T., Schiesser, J. V., Cameron, F., Mannering, S.I., Elefanty, A.G., and Stanley, E.G. (2019). Induced pluripotent stem cell macrophages present antigen to proinsulin-specific T cell receptors from donor-matched islet-infiltrating T cells in type 1 diabetes. Diabetologia *62*, 2245–2251.

Kong, W., Fu, Y.C., and Morris, S.A. (2020). Capybara: A computational tool to measure cell identity and fate transitions. BioRxiv 2020.02.17.947390.

Mancuso, R., Van Den Daele, J., Fattorelli, N., Wolfs, L., Balusu, S., Burton, O., Liston, A., Sierksma, A., Fourne, Y., Poovathingal, S., et al. (2019). Stem-cell-derived human microglia transplanted in mouse brain to study human disease. Nat. Neurosci. *22*, 2111–2116.

Ng, E.S., Davis, R., Stanley, E.G., and Elefanty, A.G. (2008). A protocol describing the use of a recombinant protein-based, animal product-free medium (APEL) for human embryonic stem cell differentiation as spin embryoid bodies. Nat. Protoc. *3*, 768–776.

Seabold, S., and Perktold, J. (2010). Statsmodels: Econometric and Statistical Modeling with Python.

Szklarczyk, D., Gable, A.L., Lyon, D., Junge, A., Wyder, S., Huerta-Cepas, J., Simonovic, M., Doncheva, N.T., Morris, J.H., Bork, P., et al. (2019). STRING v11: protein–protein association networks with increased coverage, supporting functional discovery in genome-wide experimental datasets. Nucleic Acids Res. *47*, D607–D613.

Turlach, B.A., and Weingessel, A. (2019). quadprog: Functions to Solve Quadratic Programming Problems. R package version 1.5-8.

Villani, A.C., Satija, R., Reynolds, G., Sarkizova, S., Shekhar, K., Fletcher, J., Griesbeck, M., Butler, A., Zheng, S., Lazo, S., et al. (2017). Single-cell RNA-seq reveals new types of human blood dendritic cells, monocytes, and progenitors. Science (80-.). *356*.

Virtanen, P., Gommers, R., Oliphant, T.E., Haberland, M., Reddy, T., Cournapeau, D., Burovski, E., Peterson, P., Weckesser, W., Bright, J., et al. (2019). SciPy 1.0--Fundamental Algorithms for Scientific Computing in Python. Nat. Methods *17*, 261–272.

Yanagimachi, M.D., Niwa, A., Tanaka, T., Honda-Ozaki, F., Nishimoto, S., Murata, Y., Yasumi, T., Ito, J., Tomida, S., Oshima, K., et al. (2013). Robust and Highly-Efficient Differentiation of Functional Monocytic Cells from Human Pluripotent Stem Cells under Serum- and Feeder Cell-Free Conditions. PLoS One *8*, e59243.
